# A New Model for Caries Risk Prediction in Teenagers Using a Machine Learning Algorithm Based on Environmental and Genetic Factors

**DOI:** 10.3389/fgene.2021.636867

**Published:** 2021-03-11

**Authors:** Liangyue Pang, Ketian Wang, Ye Tao, Qinghui Zhi, Jianming Zhang, Huancai Lin

**Affiliations:** ^1^Guangdong Provincial Key Laboratory of Stomatology, Department of Preventive Dentistry, Guanghua School of Stomatology, Hospital of Stomatology, Sun Yat-sen University, Guangzhou, China; ^2^Foshan Stomatology Hospital, School of Stomatology and Medicine, Foshan University, Foshan, China

**Keywords:** caries, risk prediction model, preventive dentistry, biomarkers, biomedical informatics

## Abstract

Dental caries is a multifactorial disease that can be caused by interactions between genetic and environmental risk factors. Despite the availability of caries risk assessment tools, caries risk prediction models incorporating new factors, such as human genetic markers, have not yet been reported. The aim of this study was to construct a new model for caries risk prediction in teenagers, based on environmental and genetic factors, using a machine learning algorithm. We performed a prospective longitudinal study of 1,055 teenagers (710 teenagers for cohort 1 and 345 teenagers for cohort 2) aged 13 years, of whom 953 (633 teenagers for cohort 1 and 320 teenagers for cohort 2) were followed for 21 months. All participants completed an oral health questionnaire, an oral examination, biological (salivary and cariostate) tests, and single nucleotide polymorphism sequencing analysis. We constructed a caries risk prediction model based on these data using a random forest with an AUC of 0.78 in cohort 1 (training cohort). We further verified the discrimination and calibration abilities of this caries risk prediction model using cohort 2. The AUC of the caries risk prediction model in cohort 2 (testing cohort) was 0.73, indicating high discrimination ability. Risk stratification revealed that our caries risk prediction model could accurately identify individuals at high and very high caries risk but underestimated risks for individuals at low and very low caries risk. Thus, our caries risk prediction model has the potential for use as a powerful community-level tool to identify individuals at high caries risk.

## Introduction

Permanent teeth caries was the most common chronic disease worldwide in 2016. A previous study reported that the global cost of dental diseases exceeded 540 billion dollars in 2015 and resulted in major health and financial burdens (Righolt et al., 2018). Therefore, there is an urgent need for effective caries control.

Accumulating evidence has shown a skewed distribution of caries; the majority of the disease was suffered by the minority teenagers in the population (Kaste et al., 1996). The conference of National Institutes of Health Consensus Development Conference Statement (2001) concluded that a focus on high-risk individuals was required for the prevention and control of dental caries (2001). Since caries is largely preventable, risk prediction models for early and accurate identification of teenagers at high risk of caries would be useful tools for designing more cost-effective caries control measures.

As a prerequisite for implementing minimally invasive treatment programs, caries risk prediction models (CRPMs) have huge potential in improving patient care because they allow individuals to choose appropriate non-invasive or invasive interventions (Domejean et al., 2017). There are four commonly used standardized caries risk assessment models at present: ADA (American Dental Association), CAT (Caries-Risk Assessment Tool), CAMBRA (Caries Management by Risk Assessment), and Cariogram. All these models included only environmental factors such as socio-demographic indicators, behavioral factors, plaque index, the number of *Streptococcus mutans*, and *Lactobacillus*, saliva flow, and salivary buffer capacity (Petersson and Twetman, 2015). Cariogram, one of the better CRPMs, has provided reliable results for few tests in children, but there is not enough evidence to prove its effectiveness in caries assessment and prediction. Cagetti et al. (2018) reported that the sensitivity of Cariogram in different samples ranged from 41.0 to 75.0%, while the specificity ranged from 65.8 to 88.0%.

Dental caries is a multifactorial disease caused by complex interactions between genetic and environmental risk factors. Environmental risk factors for caries included sugar-rich diet, poor oral hygiene, dental plaque, high numbers of cariogenic bacteria, inadequate salivary flow and so on (Selwitz et al., 2007). Genetic contribution to caries risk score variation has been reported to be 49.1–62.7% (Haworth et al., 2020). As a genetically complex phenotype, caries risk may be influenced by many loci with small contributions individually. These genetic factors that contribute to caries may include variants in loci for enamel formation, immune response, saliva, taste, and dietary habits (Vieira et al., 2014). Enamel formation was tested as being potentially involved in caries susceptibility. Patir et al. (2008) reported an association between enamelin (*ENAM*) and higher caries experience. Additionally, a relationship between the genetic variation of tuftelin (*TUFT1*) and caries could be detected only when the *Streptococcus mutans* levels were high (Slayton et al., 2005).

Therefore, CRPMs based on environmental factors alone may lead to the loss of useful information. Previous studies have suggested that constructing a disease risk prediction model with both environmental and genetic factors can stratify the disease risk more accurately than either of these factors alone (Li et al., 2019; Okubo et al., 2020). Accordingly, research is needed to construct CRPMs based on both genetic and environmental risk factors and evaluate their abilities to predict caries risk better. Thus, this prospective study aimed to construct a new CRPM including both genetic and environmental risk factors in teenagers of the Chinese population.

## Materials and Methods

### Study Population

This study was approved by the Ethics Committee of the Guanghua School of Stomatology, Sun Yat-sen University (ERC- [2018]01). The analysis consisted of two cohorts that began from March to April 2018 and were followed up for 21 months until the end, from December 2019 to January 2020, in Foshan, southern China. The two cohorts included 710 and 345 teenagers aged 13–14 years. Cohort 1 was used to construct the model, which included teenagers from two urban and two rural schools. Cohort 2 was used to evaluate the caries risk prediction model and included teenagers from one urban and one rural school. All participants completed an oral health questionnaire, clinical examination, and donated saliva samples at baseline. Written informed consent was obtained from the guardians of every participant before the study.

### Oral Health Questionnaire

Under the guidance of their guardians, the adolescents completed a well-designed oral health questionnaire consisting of three parts: Part 1 was mainly about demographic information, Part 2 was mainly about socioeconomic information, and Part 3 was mainly about oral health-related behaviors (Wang et al., 2020a). The specific variables are as follows:

The variables in part 1: sex, age, residence, whether the child is an only child in his/her family, and his/her primary caregiver.

The variables in part 2: family income, caregivers’ education levels, and whether they have dental insurance.

The variables in part 3: frequency of tooth brushing, flossing or mouthwash habits, toothpaste containing fluoride or not, professional application of fluoride, frequency of snack consumption, sweet drink consumption, and attendance in a dental clinic in the past 6 months.

### Clinical Examination

Plaque index (PlI) was evaluated using Silness and Löe’s scale (Loe, 1967), and six dental indices were recorded. Plaque samples were collected with sterile swabs, according to the procedural instructions of the cariostat kit (GangDa Medical Technology Co. Ltd., Beijing, China). The swabs were then immersed in culture media in ampules and incubated at 37°C for 48 h. Finally, the color of the medium was compared with the reference colors in the color chart provided by the cariostat kit.

After air drying, each tooth was examined and recorded as decayed, missing, or filled (DMFT). The caries status was evaluated according to the International Caries Detection and Assessment System (ICDAS) criteria (Pitts and Ekstrand, 2013). Codes 3–6 in the ICDAS system were recorded as decayed teeth. We also recorded filled and missing teeth due to caries. Oral examinations were conducted at both the baseline and after 21 months in the classrooms.

The students rinsed their mouths before the collection of unstimulated saliva. Unstimulated saliva was collected for 15 min. Students were first asked to swallow all the saliva in the mouth, then spit all the saliva into the scaled tube every 3 min and five times in total. The saliva flow rate (ml/min) was calculated, and saliva buffering capability was measured according to the Ericsson method. One milliliter of saliva was added to 3 ml of 3.3 mmol HCl within 5 min after collection and then allowed to stand for 20 mins. The final pH of the saliva was evaluated by an electrical pH meter (Wang et al., 2020b).

### Selection of Candidate Genetic Markers and DNA Analysis

Single nucleotide polymorphisms (SNPs) were selected based on the results of previous studies on caries susceptibility (*n* = 4) and screening of tag SNPs (*n* = 19). We used a candidate gene approach or related-pathway strategies to screen tag SNPs. Caries-related pathway genes, such as those involved in enamel formation, immune responses, saliva secretion, and taste, were identified based on the pathogenesis of caries. The tag SNPs were screened as described in our previous study (Wang et al., 2020b). Thus, 23 target SNPs were detected in all study participants (Table 1).

**TABLE 1:** Candidate genetic markers evaluated in this study.

Gene	Chromosome	Marker public ID	Base pair exchange (MAF)	Most severe consequence
**Enamel formation genes**				
*ENAM*	4	rs12640848	A/G (0.33)	Intron variant
		rs3796703	C/T (0.01)	Missense(leu)
*AMBN*	4	rs13115627	A/G (0.30)	Intron variant
*AMELX*	X	rs946252	C/T (0.31)	Intron variant
*TFIP11*	22	rs134143	T/C (0.35)	Non-coding transcript exon variant
		rs2097470	C/T (0.29)	Intron variant
*MMP20*	11	rs1612069	G/T (0.48)	Intron variant
		rs1784418	C/T (0.42)	Intron variant
*TUFT1*	1	rs17640579	A/G (0.22)	Intron variant
		rs3790506	G/A (0.25)	Intron variant
**Immune response genes**				
*DEFB1*	8	rs11362	C/T (0.40)	5 prime UTR variant
		rs1800972	G/C (0.14)	5 prime UTR variant
*LTF*	3	rs4547741	C/T (0.07)	Intron variant
		rs1126478	C/T (0.37)	Missense variant
*MBL2*	10	rs1800450	C/T (0.12)	Missense variant
		rs11003125	G/C (0.31)	Intron variant, upstream variant 2 KB
*MASP2*	1	rs10779570	T/G (0.36)	Intron variant
**Water channel protein gene**				
*AQP5*	12	rs1996315	G/A (0.43)	Intron variant, upstream variant 2 KB
		rs923911	C/A (0.22)	Intron variant, upstream variant 2 KB
**Saliva secretion gene**				
*CA6*	1	rs2274327	C/T (0.27)	Intron variant, missense
**Taste gene**				
*TAS1R2*	1	rs35874116	T/C (0.27)	Missense variant
		rs9701796	C/G (0.20)	missense variant
*TAS2R38*	7	rs713598	G/C (0.50)	Missense variant

From each participant, 2 ml of unstimulated saliva samples were collected and stored in Oragene DNA Self-Collection kits (Lang Fu, China) at room temperature until they were processed. Genomic DNA was extracted from saliva samples according to the manufacturer’s instructions. DNA samples were first purified using MassARRAY Nanodispenser (Sequenom, United States) and then transferred to a SpectroCHIP (Sequenom, United States) chip. Finally, the SNP markers were sequenced by matrix-assisted laser desorption/ionization time-of-flight mass spectrometry (MALDI-TOF MS) (Pang et al., 2017). First, 10 ng of genomic DNA were amplified by PCR in a final volume of 0.5 μL containing locus-specific primers at a final concentration of 10 μmol/L using 0.1-unit HotStarTaq DNA polymerase (Qiagen, Hilden, Germany). PCR conditions were 94°C for 3 min for hot start followed by 40 cycles of denaturation at 94°C for 30 s, annealing at 56°C for 25 s, and extension for 30 s at 72°C, and, finally, incubation at 72°C for 3 min. Then, PCR products were treated with shrimp alkaline phosphatase (Amersham, Freiburg, Germany) for 40 min at 37°C to remove excess deoxynucleotide triphosphates followed by 5 min at 85°C to inactivate shrimp alkaline phosphatase. Base extension reaction conditions were 94°C for 30 s followed by 40 cycles of 94°C for 5 s, 52°C for 5 s, and 80°C for 5 s, and, finally, incubation at 72°C for 3 min. The final base extension products were treated with SpectroCLEAN resin (Sequenom) to remove salts from the reaction buffer. A total of 10 nl of the reaction solution was dispensed onto a 384 format SpectroCHIP microarray (Sequenom, SanDiego, CA). The MassARRAY Analyzer Compac was used for data acquisitions from the MassARRAY SpectroCHIP. Genotyping calls were made in real-time with the Mass Array RT software (Sequenom) (Pang et al., 2020).

### Statistical Analysis

Data of all teenagers in cohort 1 were used to construct a CRPM with random forest, and those of teenagers from cohort 2 were used to verify this newly constructed model. The logistic regression model was used as a reference for performance evaluation. When we analyzed the variables associated with the occurrence and development of caries, the independent variable included the environmental variables and SNPs. The dependent variable was DMFT increment (ΔDMFT) over 21 months of follow-up, which is the outcome of this study. A previous study conducted by Chaffee BW (Chaffee et al., 2015) found that the DMFT increment was about 1.01 in the low caries risk groups after 18 months of follow-up. Remember that individuals with DMFT increments of no more than one caries after 21 months of follow-up should be classified in the low caries risk group. Chi-square tests were used to identify SNPs associated with increased risk of caries, and univariate logistic analysis was used to select environmental factors associated with caries. Variables with *P* < 0.1 were considered statistically significant and used as predictors in the caries risk prediction model. R 3.6.1 software was used to construct the model. Using the data of the training cohort (cohort1), the random forest package was used to train the random forest model, and the nTree and mtry parameters were debugged. The random forest prediction model was the most effective when nTree = 300 and mtry = 2. In the model constructed with cohort 1, we segmented the population into five different caries risk layers based on the 5-quantiles: very low, low, moderate, high, and very high caries risk. Then, we stratified the caries risk in the cohort 2 (testing cohort) population based on the cutoff value in cohort 1. The discrimination ability of the model was evaluated using receiver operator characteristic (ROC) curve analysis. The calibration ability of the model was measured via a risk stratification plot, which was used to demonstrate the similarity of the predicted absolute risk to the absolute observed risk at different risk levels.

## Results

### Characteristics of Study Samples

**FIGURE 1:**
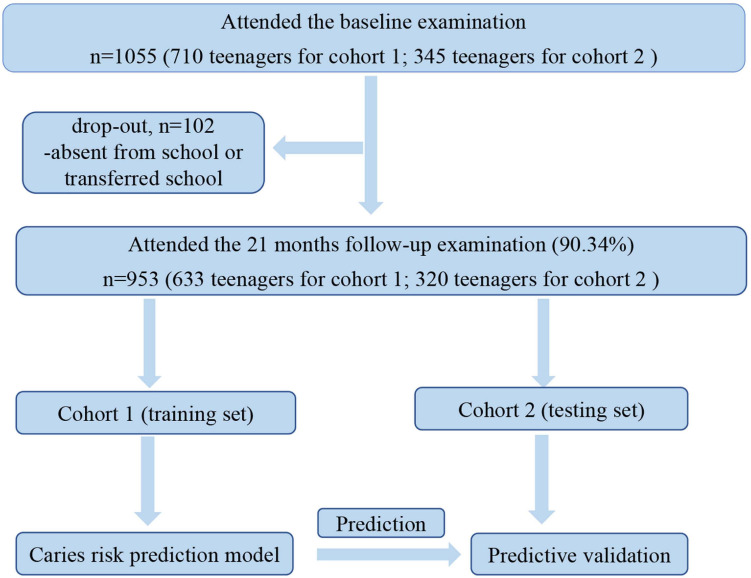
Flow chart of the prospective longitudinal study.

In total, 1,055 teenagers (710 in cohort 1 and 345 in cohort 2) were recruited. The average age at baseline was 13.19 ± 0.40 years (Wang et al., 2020a). The questionnaire was completed by all teenagers. After 21 months, 953 teenagers (including 633 teenagers in cohort 1 and 320 teenagers in cohort 2) were followed up. During these 21 months, follow-up was lost for only 102 (9.66%) teenagers. The main reasons for loss of follow-up were absence in school or transfer from schools. The flow chart of the prospective longitudinal study is shown in Figure 1.

At baseline, 34.37% of the teenagers in cohort 1 and 39.88% of those in cohort 2 were affected by caries, and the mean (SD) DMFTs were 0.67 ± 1.25 and 0.84 ± 1.38, respectively. After 21 months, 57.66% of the teenagers in cohort 1 and 63.13% of those in cohort 2 developed more than one caries (ΔDMFT > 1). The mean (SD) increases in DMFTs after 21 months were 2.40 ± 2.97 in cohort 1 and 2.73 ± 3.21 in cohort 2.

### Caries Risk Prediction Factors

Table 2 shows the results of a logistic analysis of the association between environmental variation and caries. Among the environmental variations, we found that “sex,” “dental attendance in the past 6 months,” “cariostat score,” and “past caries experience” were significantly associated with the caries risk (all *P* < 0.05).

**TABLE 2:** Logistic analysis of the association between environmental factors and caries.

Characteristics	Level	ΔDMFT ≤ 1 (*n* = 328)	ΔDMFT > 1 (*n* = 305)	*P*-value
Pit and fissure sealant (%)	No	320 (97.6)	296 (97.0)	0.879
	Yes	8 (2.4)	9 (3.0)	
Sex (%)	Female	118 (36.0)	135 (44.3)	0.041*
	Male	210 (64.0)	170 (55.7)	
Frequency of tooth brushing (%)	<1 times/day	7 (2.1)	6 (2.0)	0.127
	1 times/day	146 (44.5)	112 (36.7)	
	2 times/day	175 (53.4)	187 (61.3)	
Toothpaste (%)	No	1 (0.3)	2 (0.7)	0.95
	Yes	327 (99.7)	303 (99.3)	
Mouthwash (%)	No	243 (74.1)	230 (75.4)	0.771
	Yes	85 (25.9)	75 (24.6)	
Dental flossing (%)	No	301 (91.8)	288 (94.4)	0.247
	Yes	27 (8.2)	17 (5.6)	
Professional application of fluoride (%)	No	313 (95.4)	294 (96.4)	0.68
	Yes	15 (4.6)	11 (3.6)	
Dental attendance in the past 6 months (%)	No	166 (50.6)	122 (40.0)	0.009*
	Yes	162 (49.4)	183 (60.0)	
One-child family (%)	No	250 (76.2)	252 (82.6)	0.059*
	Yes	78 (23.8)	53 (17.4)	
Activity (%)	No	108 (32.9)	107 (35.1)	0.625
	Yes	220 (67.1)	198 (64.9)	
Cariostat score (%)	Low	85 (25.9)	48 (15.7)	<0.001*
	Medium	198 (60.4)	183 (60.0)	
	High	45 (13.7)	74 (24.3)	
Plaque Index (%)	Low	31 (9.5)	23 (7.5)	0.057*
	Medium	119 (36.3)	139 (45.6)	
	High	178 (54.3)	143 (46.9)	
Residence (%)	Urban	171 (52.1)	151 (49.5)	0.561
	Rural	157 (47.9)	154 (50.5)	
Toothpaste (%)	Non-fluoride	79 (24.1)	91 (29.8)	0.123
	Fluoride	249 (75.9)	214 (70.2)	
Saliva buffering capability (pH) (%)	PH < 3.5	94 (28.7)	94 (30.8)	0.895
	PH 3.5–4.24	104 (31.7)	89 (29.2)	
	PH 4.25–4.75	50 (15.2)	48 (15.7)	
	PH > 4.75	80 (24.4)	74 (24.3)	
Dental insurance (%)	No	251 (76.5)	230 (75.4)	0.814
	Yes	77 (23.5)	75 (24.6)	
Caregiver (%)	Mother	194 (59.1)	192 (63.0)	0.151
	Father	48 (14.6)	28 (9.2)	
	Grandparents	17 (5.2)	11 (3.6)	
	Nursemaid	11 (3.4)	8 (2.6)	
	No regular caregiver	58 (17.7)	66 (21.6)	
Education of caregiver (%)	<9 years	293 (89.3)	272 (89.2)	1
	≥9 years	35 (10.7)	33 (10.8)	
Household monthly income (CNY) (%)	<3,000	54 (16.5)	48 (15.7)	0.97
	3,000–7,000	192 (58.5)	180 (59.0)	
	≥7,000	82 (25.0)	77 (25.2)	
Frequency of snacks consuming (%)	<1 per day	215 (65.5)	211 (69.2)	0.374
Saliva secretion(ml/min)	≥1 per day <0.1 0.1–0.25 >0.25	113 (34.5) 31 (9.5) 62 (18.9) 235 (71.6)	94 (30.8) 33 (10.8) 60 (19.7) 212 (69.5)	0.801
Frequency of sweet drinks consuming (%)	<1 per day	212 (64.6)	193 (63.3)	0.786
	≥1 per day	116 (35.4)	112 (36.7)	
Past caries experience (%)	No	273 (83.2)	170 (55.7)	<0.001*
	Yes	55 (16.8)	135 (44.3)	

ΔDMFS, mean increment of decayed, missing, or filled surfaces over 21 months. Past caries experience means whether the individual had caries at the baseline examination or not. Univariate logistic regression was used to analyze the environmental factors related to the occurrence and development of caries. *P < 0.1.

Table 3 shows the results of the chi-square tests on the association between SNPs and caries. Among all the SNPs, rs1996315 (*AQP5*), and rs3790506 (*TUFT1*) were significantly associated with caries risk (all *P* < 0.05).

**TABLE 3:** Chi-square test analysis of the association between SNPs and caries.

		ΔDMFT ≤ 1			ΔDMFT>1					
SNP	Allele 1/2	11	12	22	11	12	22	OR	95% CI	*P*-value
rs10779570	G/T	21	111	196	17	105	183	0.97	0.75–1.26	0.824
rs11003125	C/G	55	173	100	48	170	87	1.02	0.81–1.29	0.860
rs1126478	C/T	163	133	32	144	121	40	1.15	0.91–1.45	0.231
rs11362	C/T	121	161	46	115	138	52	1.06	0.85–1.33	0.604
rs12640848	A/G	219	95	14	198	94	13	1.07	0.81–1.41	0.631
rs13115627	A/G	190	118	20	175	120	10	0.93	0.71–1.21	0.578
rs134143	T/C	152	132	44	129	140	36	1.05	0.83–1.31	0.699
rs1612069	G/T	84	177	67	77	176	52	0.93	0.74–1.18	0.567
rs17640579	A/G	176	133	19	156	121	28	1.17	0.91–1.5	0.214
rs1784418	C/T	95	168	65	77	169	59	1.07	0.85–1.35	0.548
rs1800450	C/T	239	82	7	235	63	7	0.85	0.61–1.16	0.305
rs1800972	G/C	260	60	8	240	63	2	0.93	0.66–1.31	0.671
rs1996315	G/A	110	160	58	116	154	35	0.79	0.62–0.99	0.042*
rs2097470	C/T	170	136	22	150	139	16	1.02	0.79–1.32	0.858
rs2274327	C/T	162	139	27	140	141	24	1.07	0.84–1.37	0.579
rs35874116	C/T	4	58	266	0	77	228	1.31	0.92–1.89	0.138
rs3790506	G/A	187	125	16	158	114	33	1.33	1.04–1.71	0.024*
rs3796703	C/T	309	15	4	287	13	5	1.06	0.64–1.76	0.830
rs457741	C/T	293	32	3	277	28	0	0.76	0.46–1.25	0.283
rs713598	C/G	30	137	161	31	125	149	1.03	0.81–1.31	0.811
rs923911	C/A	199	116	13	201	87	17	0.90	0.69–1.17	0.434
rs946252	C/T	136	54	138	127	66	112	0.93	0.79–1.11	0.440
rs9701796	C/G	204	112	12	194	99	12	0.97	0.74–1.29	0.857

ΔDMFS, mean increment of decayed, missing, or filled surfaces over 21 months. Chi-square test was used to analyze the SNPs related to the occurrence and development of caries. *P < 0.05.

### CRPM Training and Validation

**FIGURE 2:**
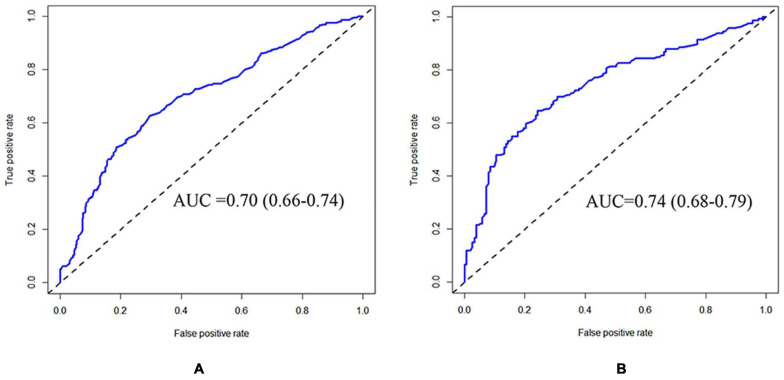
ROC curve of training and testing cohort (Logistic Regression Model). Measurement of the discrimination ability of the caries risk prediction model (Logistic Regression) with ROC curve. The AUC (95%CI) of the training cohort was 0.70 (0.66–0.74) **(A)**, and the AUC (95% CI) of the testing cohort was 0.74 (0.68–0.79) **(B)**.

**FIGURE 3:**
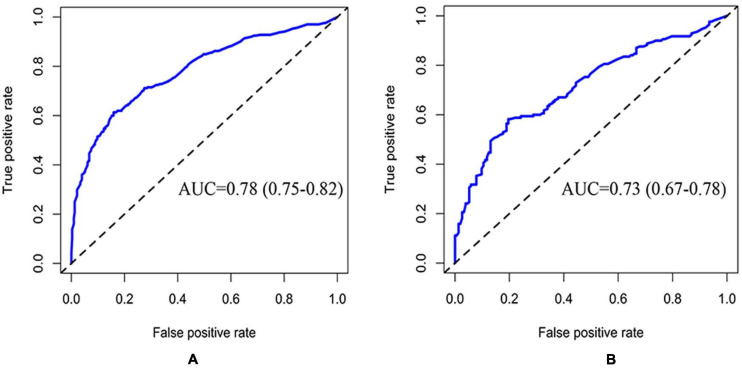
ROC curve of training and testing cohort (Random Forest Model). Measurement of the discrimination ability of the caries risk prediction model (Random Forest) with ROC curves. The AUC of the training cohort was 0.78 (0.75–0.82) **(A)**, and the AUC of the testing cohort was 0.73 (0.67–0.78) **(B)**.

The CRPM has been developed using logistic regression and random forest. The performance of CRPM developed using logistic regression was 0.70 (0.66–0.74) for the training cohort (Figure 2A) and 0.74 (0.68–0.79) for the test cohort (Figure 2B). The performance of the random forest was 0.78 (0.75–0.82) for the training cohort (Figure 3A) and 0.73 (0.67–0.78) for the test cohort (Figure 3B). The results showed that the prediction performance of the CRPM constructed using Random Forest was stable.

**FIGURE 4:**
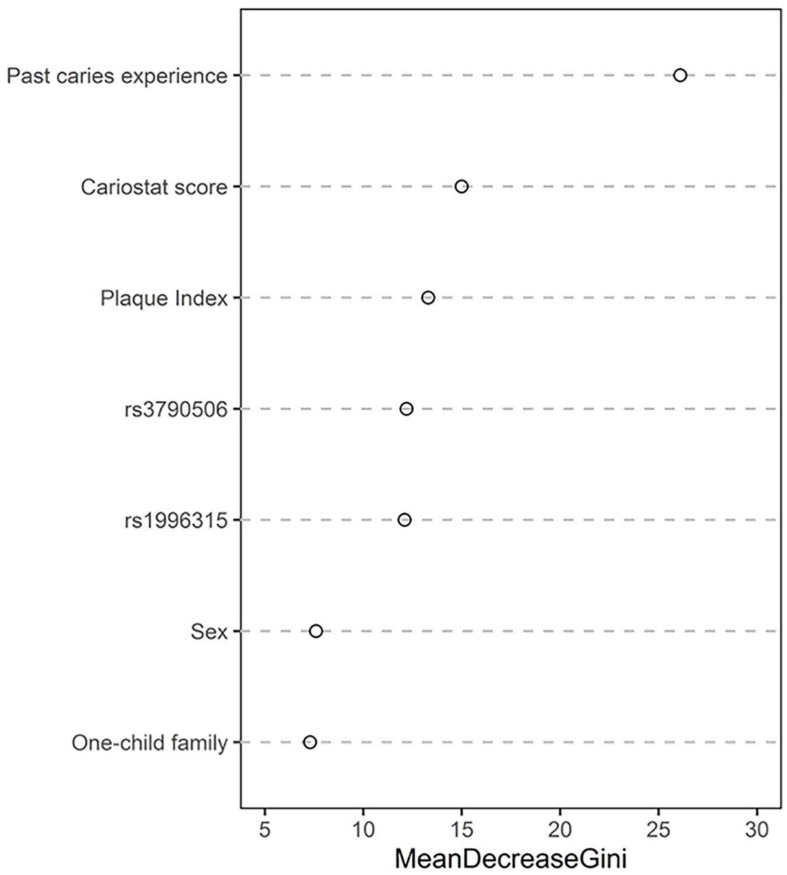
The Gini coefficient of the random forest.

The Gini coefficient of the random forest suggested that the selected variables in this prediction model could be arranged as follows according to their importance: “past caries experience,” “cariostate score,” “plaque index,” “rs3790506,” “rs1996315,” “gender,” and “whether they were only teenagers” (Figure 4).

**FIGURE 5:**
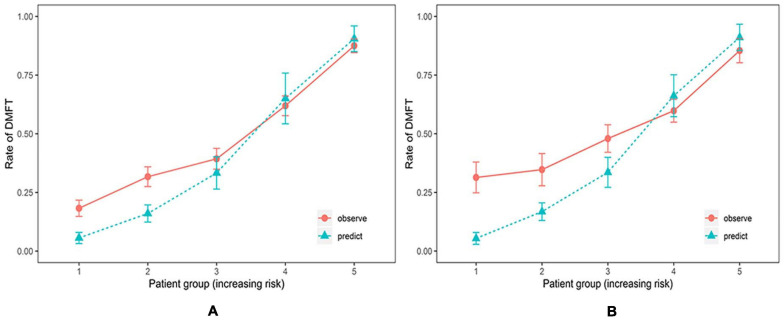
Risk stratification plot for the training and testing cohort (Random Forest Model). Relationship between observed (orange, 95% confidence intervals) and predicted (green) scores of new carious lesions for 21 months for the training cohort **(A)** and the testing cohort **(B)**. The prediction model could accurately estimate risk for individuals at high and very high caries risk but underestimated risks for individuals at low and very low caries risk.

The ability of the CRPM to identify caries risk in individuals was examined further. A risk stratification plot was created, in which the data from 320 patients in cohort 2 were sorted by increasing the predicted risk and separated into five risk layers: very low, low, medium, high, and very high. Then, the actual rate of caries incidence after 21 months was calculated for each risk layer. Figure 5 shows the degree of discrepancy between the actual and predicted risks for each of the five risk layers.

Using the CRPM constructed with the training cohort, we assigned the participants in cohort 1 into five risk groups based on the 5-quantiles of the predicted incidence probabilities as follows: very low, low, medium, high, and very high. The predicted incidence rates of caries after 21 months in cohort 1 for each risk layer were 5.60, 16.02, 33.29, 65.06, and 90.51%, respectively, and the actual incidence rates of caries after 21 months in cohort 1 for each risk layer were 18.25, 31. 71, 39. 34, 61. 94, and 87.50%, respectively (Table 4). The numbers of individuals in the caries layers of cohort 2, i.e., very low, low, medium, high, and very high, were 48,49,73,102, and 48, respectively, and the mean DMFT increment in each risk layer are shown in Table 5; the predicted incidence rates of caries after 21 months in each risk layer of cohort 2 were 5.41, 16.79, 33.56, 66.20, and 91.07%, respectively, and the actual incidence rates of caries after 21 months in each risk layer of cohort 2 were 27.08, 34.69, 47.95, 59.80, and 85.42%, respectively (Table 5). The risk of new caries was consistently reduced from the extremely high-risk category to the extremely low-risk category, reflecting the ability of our newly constructed CRPM to estimate future caries accurately.

**TABLE 4:** Actual number of new caries after 21 months: actual and predicted caries incidences in cohort 1.

Caries risk	Total number of participants in cohort 1 (n)	Actual number of new caries incidence in cohort 1 (n)	Actual caries incidence in cohort 1 (%)	Predicted caries incidence in cohort 1 (%)
Very low	126	23	18.25	5.60
Low	123	39	31. 71	16.02
Moderate	122	48	39. 34	33.29
High	134	83	61. 94	65.06
Very high	128	112	87. 50	90.51

**TABLE 5:** Actual number of new caries after 21 months: actual and predicted caries incidences in cohort 2.

Caries risk	Total number of participants in cohort 2 (n)	Actual number of new caries incidence in cohort 2 (n)	Actual caries incidence in cohort 2 (%)	Predicted caries incidence in cohort 2 (%)	Caries increment mean (SD)
Very low	48	13	27.08	5.41	1.25 ± 2.12
Low	49	17	34.69	16.79	1.67 ± 2.63
Moderate	73	35	47.95	33.56	2.39 ± 2.93
High	102	61	59.80	66.20	3.43 ± 3.72
Very high	48	41	85.42	91.07	4.33 ± 2.90

The sensitivity, specificity, positive predictive value, and negative predictive value of cohorts 1 and 2 are displayed in Table 6. The positive predictive value was high (>73%) for those stratified into very high and high caries risk categories. When the “moderate caries risk” and “low caries risk “categories were used as a cutoff level, the negative predictive values were low.

**TABLE 6:** Sensitivity, specificity, and predictive values for new caries lesions over 21 months.

Caries risk	Sensitivity (%)		Specificity (%)		PPV^a^ (%)		NPV^b^ (%)		Youden’s index^c^	
	Cohort 1	Cohort 2	Cohort 1	Cohort 2	Cohort 1	Cohort 2	Cohort 1	Cohort 2	Cohort 1	Cohort 2
Very-high	67.8	65.8	75.0	57.2	95.0	90.0	25.0	22.2	0.43	0.23
High	54.2	59.0	68.7	68.3	73.8	73.5	47.9	52.8	0.23	0.27
Moderate	45.8	34.3	69.0	65.8	48.9	48.0	66.2	52.1	0.15	0.001
Low	41.0	29.4	73.9	62.5	42.1	29.4	72.9	62.5	0.15	0.08

*^*a*^PPV, positive predictive value. ^*b*^NPV, negative predictive value. ^*c*^Youden’s index, sensitivity + specificity − 1.*

## Discussion

In this study, a new caries risk prediction model was constructed, using both environmental risk factors, such as cariostate score, plaque index, and past caries experience, and genetic factors as predictors. To our knowledge, this is the first CRPM constructed with both environmental and genetic factors, using machine learning algorithms. We further verified the accuracy of this prediction model using another independent cohort, and the results demonstrated that this CRPM could effectively identify high caries-risk individuals.

It is well recognized that dental caries is a multifactorial disease. Environmental and genetic factors play important roles in the occurrence and development of caries (Yildiz et al., 2016). Combining genetic factors with environmental factors to explain the incidence of caries is both reasonable and necessary. Being a polygenetic disease, caries is difficult to predict based on a single SNP or SNPs of individual genes. Hence, it is necessary to select SNPs from different candidate genes. In this study, SNPs were selected based on the results of previous studies, combining tag SNP screening via related-pathway strategies and candidate gene approach (Opal et al., 2015). Finally, 23 SNPs from 16 candidate genes were included in this study. After analyzing the correlation of each SNP, two SNPs were found to be associated with caries in the Chinese population.

The SNPs included in the final CRPM described here were rs3790506 and rs1996315. Of these, rs3790506 is an SNP of *TUFT1*, which is involved in enamel development and mineralization. Previous studies have reported a relationship between *TUFT1* and caries incidence in both children and adults. Slayton et al. suggested that rs3790506 in *TUFT1* interacts with the *Streptococcus mutans* present in the oral cavity and further explained over a quarter of the factors affecting the variability of caries conditions in teenagers from Iowa, United States (Slayton et al., 2005). rs1996315 is a SNP of *AQP5*, which encodes a water channel protein expressed in lacrimal and salivary glands and epithelial cells. Aquaporins play a role in the generation of tears, saliva, and pulmonary secretions. *AQP5* protein also plays an important role in extracellular matrix hydration during tooth development (Felszeghy et al., 2004). It has been reported that variations in *AQP5* could contribute to the occurrence and development of caries (Wang et al., 2012; Anjomshoaa et al., 2015). Our previous study showed that gene-gene interaction between rs1996315 and rs923911 was significantly associated with molar-incisor hypomineralization (Pang et al., 2020). Both SNPs included in the CRPM constructed in this study were associated with enamel development. The etiological theory of dental caries states that enamel characteristics also affect the pathogenesis of dental caries, although it is not feasible to detect the physical and chemical characteristics of enamel *in vivo*. The identification of variations in enamel-related genes can indirectly reflect enamel characteristics associated with the occurrence of dental caries. Although genetic factors were included in this CRPM, it should be noted that environmental factors were more dominant than genetic factors. Silva et al. revealed that, compared to environmental factors, genetic factors have relatively little influence on the risk of dental caries, which is consistent with the results of our study (Silva et al., 2019).

In accordance with the results of traditional CRPMs, such as the Cariogram model, the CRPM constructed in this study using a machine learning algorithm identified “past caries experience” as the strongest predictor of individual risk. Besides the “past caries experience,” “cariostate score,” “plaque index,” “gender,” and “whether they were only teenagers in the family” were also included in this new CRPM. Unlike the Cariogram model, we used the “cariostate score” instead of “bacterial counts” to evaluate the cariogenic ability of the dental plaque. Cariostat uses a colorimetric test to evaluate the acid produced by bacteria in the plaque (Ramesh et al., 2013). The occurrence of carious lesions is a dynamic process in which acids produced by bacteria impact the demineralization of dental tissues (Richards et al., 2017). When the pH of the tooth surface decreases to a level < 5.5, the hydroxyapatite (HA) matrix of the tooth starts to demineralize; Cariostat can assess the activity of the caries microbiology. Unlike other cariogenic microbiology tests, such as Dentocult SM, Cariostat assesses bacteria in plaque instead of saliva, leading to higher accuracy because cariogenic bacteria act on tooth surfaces in the form of plaque.

An ideal but possibly unrealistic model will correctly distinguish individuals at risk of a caries event from those who are not at risk, without any instance of misdiagnosis (Alba et al., 2017). The extent to which a model can achieve this goal is represented by two related properties of discrimination and calibration (Alba et al., 2017). Discrimination refers to the extent to which the model distinguishes between high-risk and low-risk participants of an event, usually described by the receiver operating characteristic (ROC) curve. It is well recognized that an AUC < 0.6 represents poor discrimination, while an AUC ≥ 0.7 indicates high discrimination ability (Fontana et al., 2020). The training set resulted in an AUC of 0.78 in cohort 1 and 0.73 in cohort 2, indicating high discrimination ability.

Discrimination alone is not sufficient to evaluate the performance of a prediction model. The second essential characteristic of a prediction model is demonstrating the similarity of the predicted absolute risk to the absolute observed risk at different risk levels. Calibration is usually considered the most important characteristic of a prediction model because it reflects the extent to which a model correctly predicts the absolute risk (Alba et al., 2017). In terms of accurate estimation, the model is well-calibrated. The relationship between predicted and observed risk could be visually represented, allowing efficient evaluation of the calibration (Alba et al., 2017). We found that the CRPM constructed in this study can accurately estimate the risks of individuals at high and very high caries risks but underestimates those for individuals at low and very low caries risks. However, this poor calibration may not pose a problem for low-risk individuals because the purpose of this CRPM is to identify teenagers at high risk of developing caries for better prevention and intervention, and the underestimation of patients at lower risk would be rather irrelevant. Hence, our CRPM can be considered a useful tool for selecting high caries risk population in China.

Our study has several limitations. First, although the SNPs were selected based on the results of previous studies on caries susceptibility and through screening of tag SNPs from multiple genes, it cannot be ruled out that some key loci with powerful diagnostic performance were missed. As an infectious disease, caries risk will certainly be affected by microorganisms. Even if we use “cariostate score” to evaluate the cariogenic ability of the dental plaque, the prediction performance might be influenced by microbiome markers. Although the ICDAS system was used to record caries, earlier signs (ICDAS code 1 or 2) of caries were not detected in our study. In addition, despite external verification with an independent cohort, further multicenter research is also highly needed.

In conclusion, we constructed a CRPM based on both environmental and genetic factors using a machine learning algorithm. We also estimated the discrimination and calibration abilities of this CRPM using a separate independent cohort for validation, demonstrating that this CRPM can accurately identify a high caries risk population. Our CRPM included specific patient characteristics, such as SNPs, gender, and whether the participants were the only child of the respective families, to provide an estimate of the absolute risk of a specific caries outcome. Thus, our CRPM can be utilized as a powerful tool at the community level for identifying high caries risk groups, enabling policymakers to plan necessary preventive measures for the future.

## Data Availability Statement

The data presented in the study are deposited in the European Variation Archive (EVA) repository, accession number PRJEB43233. The data will first be made available to download here: https://www.ebi.ac.uk/ena/data/view/PRJEB43233.

## Ethics Statement

The studies involving human participants were reviewed and approved by the Ethics Committee of the Guanghua School of Stomatology, Sun Yat-sen University (ERC- [2018]01). Written informed consent to participate in this study was provided by the participants’ legal guardian/next of kin.

## Author Contributions

LP contributed to conception, design, and drafted the manuscript. KW contributed to data acquisition, analysis, and critically revised manuscript. YT contributed to design and critically revised manuscript. QZ contributed to conception and drafted manuscript. JZ contributed to design and critically revised manuscript. HL contributed to conception, design, and critically revised manuscript. All authors gave final approval and agreed to be accountable for all aspects of the work.

## Conflict of Interest

The authors declare that the research was conducted in the absence of any commercial or financial relationships that could be construed as a potential conflict of interest. The reviewer SZ declared a past co-authorship with one of the authors HL to the handling editor.
